# Treatment of Post-Traumatic Stress Disorder Nightmares at a Veterans Affairs Medical Center

**DOI:** 10.3390/jcm5120117

**Published:** 2016-12-16

**Authors:** Mark B. Detweiler, Bhuvaneshwar Pagadala, Joseph Candelario, Jennifer S. Boyle, Jonna G. Detweiler, Brian W. Lutgens

**Affiliations:** 1 Staff Psychiatrist, Veterans Affairs Medical Center, Salem, VA 24153, USA; 2 Department Psychiatry, Edward via College of Osteopathic Medicine, Blacksburg, VA 24060, USA; 3 Virginia Tech-Carilion Clinic Psychiatry Residency Program, Roanoke, VA 24014, USA; pagadala.bhuvaneshwar@gmail.com; 4 Geriatric Research Group, Veterans Affairs Medical Center, Salem, VA 24153, USA; Joseph.Candelario@va.gov (J.C.); braciano@bellaltlantic.net (J.G.D.); blutgens@comcast.net (B.L.); 5 Emergency Department, Veterans Affairs Medical Center, Salem, VA 24153, USA; 6 Attending Physician, Veterans Affairs Medical Ce.nter, Syracuse, NY 13210, USA; Jennifer.Boyle2@va.gov

**Keywords:** veterans, post-traumatic stress disorder, nightmares, pharmacotherapy, combat, soldiers

## Abstract

The effectiveness of medications for PTSD in general has been well studied, but the effectiveness of medicatio.ns prescribed specifically for post-traumatic stress disorder (PTSD) nightmares is less well known. This retrospective chart review examined the efficacy of various medications used in actual treatment of PTSD nightmares at one Veteran Affairs Hospital. Records at the Salem, VA Veterans Affairs Medical Center (VAMC) were examined from 2009 to 2013 to check for the efficacy of actual treatments used in comparis.on with treatments suggested in three main review articles. The final sample consisted of 327 patients and 478 separate medication trials involving 21 individual medications plus 13 different medication combinations. The three most frequently utilized medications were prazosin (107 trials), risperidone (81 trials), and quetiapine (72 trials). Five medications had 20 or more trials with successful results (partial to full nightmare cessation) in >50% of trials: risperidone (77%, 1.0–6.0 mg), clonidine (63%, 0.1–2.0 mg), quetiapine (50%, 12.5–800.0 mg), mirtazapine (50%; 7.5–30.0 mg), and terazosin (64%, 50.0–300.0 mg). Notably, olanzapine (2.5–10.0) was successful (full remission) in all five prescription trials in five separate patients. Based on the clinical results, the use of risperidone, clonidine, terazosin, and olanzapine warrants additional investigation in clinically controlled trials as medications prescribed specifically for PTSD nightmares.

## 1. Introduction

### 1.1. Definition of Post-Traumatic Stress Disorder Nightmares

Active duty soldiers often have their sleep–wake cycles disrupted due to evolving combat situations in the field and chronic effects after returning home as active duty soldiers or as veterans. The increase in sympathetic arousal during combat and other life-threatening situations is responsible for many of the symptoms of post-traumatic stress disorder (PTSD), such as hyperarousal and nightmares [1]. PTSD is characterized by dysregulation of the autonomic nervous system, frequently accompanied by nightmares. PTSD is a trauma- and stress-related disorder that may develop following exposure to actual or threatened death, serious injury, sexual violence, or witnessing or learning about the death of a close family member or close friend [2]. The essential element is psychological, although physical injury may also be present. The U.S. National Comorbidity Survey Replication estimated that the lifetime prevalence of PTSD among adult Americans between February 2001 and April 2003 was 6.8% [3,4]. However, the estimated lifetime prevalence of PTSD was 30.9% for males and 26.9% for females Vietnam veterans [5], 10.1% among the total Gulf War veterans [6], and 13.8% for Iraq and Afghanistan veterans [7] all higher than for non-veteran Americans. The disease may persist for more than 20 years [8,9], with adequate treatment often shortening the years of active symptoms [10].

Some experts consider sleep disturbances the hallmark of PTSD (DSM-V [2]). They are often reported as the most intrusive and disruptive PTSD symptom [11]. PTSD nightmares are a subset of Nightmare Disorder, classified as Parasomnia in DSM-V [2]. Nightmares are dreams portraying representations of the traumatic events during sleep that are vividly recalled, creating dysphoria and anxiety due to the associated memories of a traumatic event. Nightmares most frequently occur during the latter part of a sleep episode [2]. They are associated with sudden awakening, tachycardia, diaphoresis, and fear. Combat nightmares and non-PTSD nightmares may exert a serious impact on social, occupational, personal, and marital functioning [12,13]. These sleep disturbances may be treated as a core feature rather than a secondary symptom of PTSD [14].

### 1.2. Impact of PTSD Nightmares

In combat, regular sleep–wake cycles are often not possible due to evolving combat situations. PTSD nightmares are commonly described by soldiers who participated in any recent military action. Subjective reports of sleep abnormalities among combat veterans diagnosed with PTSD revealed that 44%–90% of patients reported that they had experienced difficulties falling and staying asleep [15,16]. De Fazio et al. [17] reported that more than 60% of Vietnam combat veterans had one or more nightmares per month. In another study, recurrent nightmares were reported by 52%–87% of the veterans interviewed [18]. Among Iraqi and Afghanistan active duty soldiers in a U.S. Army Warrior Transition Clinic (WTC), sleep disturbances were cited by primary care physicians in 22% of consults and in 6% of the active duty soldiers seen by WTC telepsychiatrists on intake and continuing care [13].

### 1.3. What Is Known about the Use of Medications to Stop or Modulate PTSD Nightmares?

Multiple medications have been employed for the treatment of PTSD nightmares, with mixed success [19,20,21]. A variety of classes of medications have been prescribed for the treatment of PTSD nightmares and PTSD-related sleep disturbances, including serotonin reuptake inhibitors [22], typical antipsychotics [23], atypical antipsychotics [24], tricyclic antidepressants [25], alpha 2-adrenergic receptor agonists [26], non-benzodiazepine imidazopyridines [27], benzodiazepines [28], hypnotics [29], sulfamate-substituted monosaccharaides [30,31,32], non-selective beta blockers [33], nipecotic acid derivatives [34,35], anxiolytics [36], anticonvulsants [37], monoamine oxidase inhibitors [38,39], glucocorticoids [40,41], and others [19,20,21]. The aim of this retrospective study was to assess the effectiveness of pharmacological agents prescribed specifically to treat PTSD nightmares in one Veterans Affairs Medical Center (VAMC) and to compare these results with results cited in three combat nightmare medication reviews [19,20,21].

## 2. Methods

### 2.1. Participants

This study was approved by the Institutional Review Board and the Research Committee of the Veterans Affairs Medical Center. Search terms utilized to find patient records included “PTSD + nightmares” and “PTSD + sleep disorders”. The electronic records of 2131 veterans and active duty soldiers were initially examined from 2009 to 2010 and followed from the start of the medication prescription until completion or until November 2013 when data collection ended.

The inclusion criterion were: (1) A diagnosis of PTSD based on DSM-IV [42] and DSM-IV TR [43]; (2) clinical notes indicating that a specific medication or medications were prescribed specifically for treating nightmares associated with PTSD; (3) subsequent clinical notes indicating the response to the medication as no change, improved with decrease in frequency and severity, or total suppression of the nightmare. A medication trial consisted of the use of the medication at the same dose until the dose was increased, decreased, or stopped. A change in dose of any medication was considered a new trial for the length of the time that that dose was prescribed. If there was no formal termination of the medication by the clinician after November 2013, the length of the trial extended from the beginning of the particular dose until the last day of November 2013.

Exclusion criteria were: (1) Clinical notes that did not specifically state that a medication or medications were prescribed specifically to treat PTSD nightmares; (2) clinical notes that did not have the response to the medication prescribed for nightmares; (3) clinical notes that did not indicate when and why the medication was stopped. Treatment response was graded at three levels: no response (no change in number and/or severity of nightmares); partial response (decrease in number and/or severity of nightmares); or full response (no recall of nightmare content). Where applicable, a chi-squared analysis of response by race was used to determine if Caucasians and African Americans differed in their responses to each medication.

### 2.2. Literature Search

Literatures using MEDLINE and other International Literature in the Traumatic Stress database were reviewed from 2000 to 2014. Search terms included “PTSD”, “nightmares”, “pharmacotherapy “, “combat” and “PTSD”. The literature search was extended to before 2000 if there was no relevant information found during the literature search from 2000 to 2014.

## 3. Results

### 3.1. Participants; Department of Health and Human Services, Office of Human Research Protection (OHRP) Code of Federal Regulations Title 45, Part 46 (45 CFR 46)

The final VA sample consisted of 327 veterans and active duty soldiers with 478 cases of combat nightmares, involving 24 individual medications and 16 different medication combinations. Of the 558 cases identified in the electronic record search, 85.6% (478) were included in the analysis of nightmare medication outcomes.

Demographically, there were 313 men (95.7%), 13 women (4.0%), and one unknown (0.3%), aged 23–82 years (mean 53.7, median 61), with 242 Caucasians (74.0%), 73 African Americans (22.3%), six Hispanics (1.8%), three Native Americans (0.9%), two “other” or “unknown” (0.6%), and one Hawaiian (0.3%). One hundred and eighty-two of the sample members were married (55.7%), 105 were divorced or separated (32.1%), 31 were single (9.5%), and nine were widowed (2.8%). Where representation by race was possible, a chi-squared analysis revealed no significant difference in response between African Americans and Caucasians (*p* values ranged from 0.10 to 0.60).

### 3.2. Medication Effectiveness

The medications employed for treating nightmares were initially divided into classes. Table 1 show the medications; dose ranges; number of no, partial, and full responses; percent response for each of these three response grades; and the longest prescription length. This information was included for each of the 21 individual medications and the 13 medication combinations.

Overall, PTSD nightmares were responsive to treatment, with the individual and medication combinations having a total success rate (partial + full responses) of 56.8%. Of the single agents with 20 or more prescriptions, prazosin was prescribed most frequently (106 trials), with a success rate of 49% at a dose range of 1.0 to 10.0 mg with the longest trial of 699 days. The failure rate of 51% included an expanded dose range of 1.0 to 20.0 mg. Two other frequently utilized alpha 2 adrenergic receptor agonists were clonidine (27 trials) and terazosin (26 trials). They proved more effective than prazosin with success rates of 63.0% and 64.0%, respectively, although they were less frequently prescribed than prazosin (Table 1). Clonidine has a larger failure (0.1–4.0 mg) than success (0.1–2.0 mg) dose range, whereas for terazosin, the failure dose range was smaller (1.0–20.0 mg) than for the success (2.0–50.0 mg) dose range.

The atypical antipsychotic risperidone (81 trials) had the best success rate (77%) of all agents prescribed for more than 20 or more veterans. The success (0.25–5.0 mg) and failure (0.25–6.0 mg) dose ranges were relatively similar. Another atypical antipsychotic, quetiapine (70 to trials), was the third most prescribed anti-nightmare agent. However it had as many successes as failures. Of those medications prescribed three to five times, olanzapine (100%, range 2.5–20.0 mg), perphenazine (75%, range 4.0–8.0 mg), and ziprasidone (66.7%, range 40.0–80.0 mg) had the most favorable outcomes, while aripiprazole (25%) had the poorest outcome at a dosing range of 5.0–10.0 mg (Table 1).

Prazosin was the only alpha 2 adrenergic receptor agonist that resulted in total nightmare resolution (six cases), with one prescription lasting 699 days. Clonidine, terazosin, and diphenhydramine produced more beneficial outcomes (33) than negative outcomes (19). Both clonidine and terazosin had partial responses of more than one year (557 and 745 days, respectively).

Of the 2 medication combinations with more than 4 prescriptions, prazosin was employed in both (Table 1). Prazosin (range 2.0–10.0 mg) plus trazodone (range 50–100 mg) failed in 60% of the cases, while prazosin (range 1.0–5.0 mg) plus quetiapine (range 25–450 mg) failed in 80% of cases. Prazosin (5.0 mg) and risperidone (1.5 mg) failed in one trial of 41 days and had a full response (prazosin 1.0 mg + risperidone 2.0 mg) in a 30-day trial.

Table 2 indicates the mode dose for the seven medications with 20 or more prescriptions written. Of these, the effective dose mode was higher for quetiapine (50 mg) and terazosin (2.0 mg) than for the unsuccessful trials (25 and 1.0 mg, respectively). The effective mode dose was the same as the ineffective mode dose for clonidine (0.1 mg), prazosin 2.0 mg), risperidone (2.0 mg), and trazodone (100.0 mg). The effective mode dose for mirtazapine was lower (15.0 mg) than the ineffective mode range (30.0 mg).

Table 3 reviews the studies that demonstrated effective nightmare medication dose ranges in the three review articles (Davidson 2005 [19], Van Liempt 2006 [20], Aurora 2010 [21]) in comparison to the results of the VAMC and WTC nightmare medication prescriptions. Studies cited by the three reviewers in which there was not an effective outcome for nightmare treatment outcome were excluded from Table 3.

### 3.3. Comparison with Davidson 2005, Van Liempt 2006, and Aurora 2010 

#### 3.3.1. Alpha 2 Adrenergic Receptor Agonists—Results

In the alpha 2 adrenergic receptor agonist group of agents employed for nightmare relief, the effective dose range was similar for prazosin among the three reviews, with the range cited by Aurora et al. [21] having the highest effective dose (16.3 mg) (Table 3). Clonidine improved nightmares in one study (four cases) cited by Van Liempt et al. [20] at a dose of 0.3 mg, whereas the VAMC had nightmare improvement at lower doses (0.1–2.0 mg) in 17 cases (63%) and within a broader dosing range of 0.1–4.0. Terazosin was not mentioned in the other studies, but at the VAMC it improved nightmares in 16 cases (61%) within a dosing range of 2.0–50.0 mg. Cyproheptadine was not prescribed by the VAMC clinicians. However, in the four studies with a total of 30 patients cited in the Van Liempt et al. [20] and Aurora et al. [21] reviews, the effective dose range was 4.0–24.0 mg.

#### 3.3.2. Atypical and Typical Antipsychotics

All the atypical and typical antipsychotics except aripiprazole improved or resolved nightmares in 50% or more of the trials. In the VAMC study, risperidone and quetiapine were prescribed more frequently (81 and 72 cases, respectively) than in the other studies cited. Risperidone was successful at nightmare resolution (26%) and nightmare improvement (51%) in the majority of cases. The dosing range employed by the VA clinicians was broader (0.25–6.0 mg) than in the three successful studies cited by Van Liempt et al. [20] and Aurora et al. (0.5–3.0 mg) [21].

One successful quetiapine study was cited by Van Liempt et al. [20] in which there was nightmare improvement in a group of 20 patients receiving 25.0–300.0 mg. When prescribed at the VAMC, quetiapine resolved nightmares in six cases (8%), improved nightmares in 30 cases (42%), and failed to improve nightmares in 36 cases (50%). The maximum successful quetiapine dose was higher (800.0 mg) than the maximum dose (500.0 mg) in cases with no nightmare improvement.

Olanzapine was cited as having a benefit for nightmare treatment in two studies with a total of 24 patients. The dose range for these two studies was 5.0–20.0 mg. At the VA, olanzapine resolved nightmares in one case (5.0 mg, 105 days) and improved nightmares in four cases, having a dose range of 2.5–10.0 mg. No VAMC cases showed a lack of PTSD nightmare benefit.

Aripiprazole was cited by Aurora et al. [21] as improving nightmares in one study with five participants receiving a dose of 15.0–30.0 mg. No veterans had complete nightmare resolution. However, one experienced improvement at 10.0 mg and three had no benefit from doses of 5.0–10.0 mg.

Ziprasidone and perphenazine were not cited by the three reviewers. The VA prescribed ziprasidone for nightmares in three cases with improvement in two (40.0–80.0 mg) and a lack of response in one (40.0 mg). The typical antipsychotic perphenazine resolved nightmare recall in one case (8.0 mg) and improved nightmares in two cases (4.0–8.0 mg). There were no cases without benefit.

#### 3.3.3. Antidepressants

Only venlafaxine and trazodone were cited by Van Liempt et al. [20] and Aurora et al. [21] as having some clinical effect. Venlafaxine was not prescribed at the VA. In the one study cited by Aurora, there was no nightmare change in 340 participants receiving 37.5–300 mg doses. Two trazodone studies including 80 participants were cited in the Van Liempt 2006 [20] and Aurora 2010 [21] reviews in which nightmare improvement occurred with doses ranging from 25.0–400 mg. Trazodone was prescribed in 22 cases at the VAMC with less than ideal results. Nightmares were resolved in one 295-day case (200 mg) and improved in seven cases with a dosing range of 50–300 mg (longest trial: 542 days). However, in 14 cases, there was no improvement with the dosing range of 50–200 mg. There were no encouraging studies cited in the three reviews utilizing tricyclics. The VAMC providers prescribed only amitriptyline in one case for nightmare treatment. At a dose of 50 mg, amitriptyline provided nightmare reduction over a period of 1013 days.

In the studies cited by Van Liempt et al. [20] and Aurora et al. [21] clonazepam at 1.0–2.0 mg was unsuccessful in a trial with six participants. Clonazepam was prescribed once at the VAMC with a 1.0 mg dose yielding a partial response for 862 days. Van Liempt et al. [20] noted an unsuccessful study with 22 subjects in which temazepam was tapered from 30 mg to stop over a period of seven days in the effort to extinguish PTSD symptoms. The one use of temazepam 15.0 mg at the VA improved nightmares for 1421 days.

All three reviews cited a study that improved nightmares by employing topiramate 12.5–500.0 mg among 35 participants. The VAMC prescribed topiramate twice: 200 mg for 365 days, and 100 mg for 30 days. The first trial improved nightmares, while the second had no benefit.

## 4. Discussion

The VAMC population included in this study consisted of both active duty soldiers and a wide age range of veterans seen by a VAMC team of psychiatrists over a period of six years. Overall, the VAMC clinician prescription practices were successful in the resolution and partial reduction of nightmare symptoms in both populations. The VAMC providers had a 56.8 percent response rate using 21 individual and 13 medication combinations. The work of other authors on PTSD regarding seven medication modalities (alpha 2 adrenergic receptor agonists, atypical antipsychotics, typical antipsychotics, antidepressants, tricyclic antidepressants, benzodiazepines, and sulfamate-substituted monosaccharaide antiepileptics) primarily supported the results in this study. However, there are some significant differences from the VAMC study that contribute important insights into the clinicians’ treatments of the nightmares often associated with PTSD.

### 4.1. Alpha 2 Adrenergic Receptor Agonists

Alpha-2 agents are thought to reduce the norepinephrine response to nightmares, which in turn may reduce the arousal level when exposed to a PTSD trigger [75,76]. In the VAMC study, the alpha 2 adrenergic receptor agonists, prazosin, clonidine, and terazosin, were the second most prescribed group (159) of single nightmare medications. The two most successful alpha 2 adrenergic receptor agonists in the VAMC study were terazosin (25) and clonidine (27), which proved more effective than prazosin although they were prescribed less frequently than prazosin. Of the alpha-2 adrenergic receptor agonist studies discussed by the three PTSD medication review authors [19,20,21], prazosin, clonidine, and cyproheptadine were the most favorable. Due to the side effects, alpha 1-adrenergic antagonists are not always appropriate for persons with depression, anxiety, orthostatic hypotension, first dose syncope, tachycardia, impotence, and priapism [77].

While prazosin was the most frequently prescribed nightmare medication (106) of all the agents employed for PTSD nightmares at the VAMC, its success rate (49%) was lower at a dose range of 1.0 to 10.0 mg, while the failure rate of 51% included a larger dose range from 1.0 to 20.0 mg. This is a confounding result as higher prazosin doses have been reported to be more effective in cases when more moderate doses have produced partial responses [78,79]. The VAMC dose ranges were congruent with what was reported by Davidson et al. [19], Raskind [44], Raskind [46], Van Liempt et al. [20], Raskind [45], Taylor [80], Peskind [81], Aurora et al. [21], Raskind [26], and Taylor 2008 [47]. Even though the upper limit of the failure dosing range (20.0 mg) for the VAMC providers in their 106 prescriptions was higher than cited in the three reviews, it was less than suggested in the more recent studies of prazosin for PTSD nightmares of 25 mg/day for men, 12 mg/day for women [79], and 45 mg [78] for PTSD nightmares.

Thus, more resistant PTSD nightmare cases may necessitate dosing of 25 mg and above, indicated by the large number of prazosin failures at the VAMC when the upper dosing range limit was 20.0 mg. A 15-week randomized controlled trial of prazosin for combat trauma nightmares, sleep quality, and other PTSD symptoms in soldiers serving in Iraq and Afghanistan found effective dosing to be the following: men, midmorning 4.0 mg, bedtime 15.6 mg; women, midmorning 1.7 mg, bedtime 7.0 mg. A higher successful dosing range (25 mg/day men, 12 mg/day women) was reported for a younger cohort (avg. 30 years) [79]. A more recent two-case report of resistant PTSD nightmares successfully employed prazosin at high doses (30 mg, 45 mg) without adverse side effects [78]. The 54 (51%) prazosin failures with doses up to 20 mg in the VAMC clinics are puzzling. These cases may constitute a subgroup of veterans and solders requiring prazosin doses greater than 20.0 mg, as required for the younger Iraqi and Afghanistan soldiers in Raskind’s 2013 study [79].

According to Aurora et al. [21], clonidine had a limited effect for PTSD nightmares based on three studies [20,48,82,83], with an effective dose of 0.3 mg [48]. While clonidine did not achieve full PTSD nightmare recall cessation at the VAMC, it had a partial response in 62.9 percent of trials (17) over a dose range of 0.1 to 2.0 mg. It was ineffective in 37.1 percent of trials (10) with a broader dose range of 0.1–4.0 mg. Clonidine is a centrally active alpha-adrenergic agonist that stimulates alpha 2-adrenoreceptors in the brain stem, especially the locus ceruleus, and activates inhibitory neurons, resulting in a decrease in vasomotor tone and heart rate. Clonidine may have potential effects on the hyperarousal symptoms of PTSD, including nightmares [84]. These limited data indicate that clonidine may have a future role as a dedicated agent for PTSD nightmares, pending more investigative results.

Although terazosin was not assessed by the three review authors [19,20,21], it was utilized for PTSD nightmares in 25 trials at the VA. While it did not induce full remission of PTSD nightmares, reduction of symptoms was recorded in 64 percent of the trials (16) from a dosing range of 2.0–50.0 mg. It failed to promote any response in 36 percent (9) at a lower dosing range of 1.0–20.0 mg. VAMC results from these 25 outpatient clinical prescriptions suggest that terazosin doses of 2.0–50 mg may be effective for PTSD nightmare treatment. However, additional studies are required to understanding the role of terazosin for both veterans and combat soldiers.

Cyproheptadine is a histamine and serotonin antagonist that has been reported to improve nightmares in several case reports with a dosing range from 4 to 24 mg per day [49,50,85]. One placebo-controlled trial (PCT) with 69 participants demonstrated no improvement in nightmare symptoms after two weeks [86]. Aurora et al. [21] rated cyproheptadine as an unproven PTSD nightmare agent based on conflicting and sparse data. Cyproheptadine was not prescribed by VAMC clinicians for PTSD nightmares.

### 4.2. Atypical Antipsychotics

Antipsychotics were the most frequently prescribed class (169) of medications at the VAMC for PTSD nightmare treatment. Atypical antipsychotics have been used with some success for nightmares for more than 20 years [84,87]. In the VAMC study, the atypical antipsychotic risperidone had the best success rate (77%) of all the 21 agents prescribed for more than 327 veterans in the VAMC study.

Davidson et al. [19] cited one randomized placebo-controlled trial (RPCT) [88] and two double-blind, placebo-controlled trial (DRPCT) [89,90] studies employing risperidone for PTSD symptoms. Insomnia was improved; however, risperidone was not specifically investigated as a dedicated agent for nightmares. Van Liempt et al. [20] cited one four-case series of combat veterans that had their PTSD nightmares reduced or extinguished with risperidone 2.0 to 3.0 mg per day [52]. Aurora et al. [21] mentioned two case studies in which risperidone improved nightmares. In a six-week trial, risperidone (1.0–3.0 mg) reduced the nightmares of 17 Vietnam veterans [91]. Another study followed 10 burn patients, all of whom had their PTSD nightmares reduced by low-dose risperidone (0.5–2.0 mg) one to two nights after initiating the medication [92]. In one study, 20 mg of risperidone improved general PTSD symptoms in 15 participants in a randomized double-blind trial; however, PTSD nightmares were not the single treatment objective [93]. These results contrast with a multicenter study (247 veterans) that also did not use risperidone for PTSD nightmares; no difference was found between the risperidone and placebo groups for symptoms of PTSD in general [94].

As a dedicated nightmare suppressant, risperidone has been effective in open-label reports [95,96]. In a study of 65 veterans with PTSD-related nightmares, 84 percent of veterans responded to risperidone (0.5–2.0 mg), of whom 92.7% responded either fully (*n* = 28) or partially (*n* = 27) [95]. Confounders such as age, metabolic indices, sleep apnea, prior hospitalizations for PTSD or other psychiatric diagnoses, prior or concurrent psychiatric medication use, and substance abuse had no statistically significant associations with treatment response [95]. In a small four-participant, open-label study, low risperidone (1.0–3.0 mg) reduced or stopped the recall of three veterans and one active duty soldier [96].

The mechanisms of action for risperidone for nightmare modulation are thought to include antiserotonergic receptor antagonism and antidopaminergic activity (5-HT 2A, 5-HT7, D2) for anxiety and insomnia [87,97], and alpha-1 and αlpha-2 adrenoreceptors’ ability to decrease sympathetic outflow, resulting in improvement of anxiety, hyperarousal, and irritability symptoms [76,87,97]. Interestingly, the VAMC study success and failure dose ranges were relatively similar: 0.25–5.0 mg compared to 0.25–6.0 mg, respectively. Total cessation of PTSD nightmares occurred on the first night at a risperidone dose of 2 mg before bed. Nightmare cessation continued despite changes in concurrent antidepressants, anxiolytics, and hypnotics. No medication side effects were reported.

The atypical antipsychotic quetiapine was the third most prescribed PTSD anti-nightmare agent [95]; however, its successes equaled its number of treatment failures. As for most antipsychotics, there is limited literature supporting the use of quetiapine for PTSD nightmares. In one study, 53 veterans with PTSD accompanied by psychotic symptoms completed eight-week in-patient treatment with quetiapine (25–400 mg/day). There was a significant reduction in total and subscale scores on the Clinician-Administered PTSD Scale (CAPS), and Clinical Global Impressions Severity Scale (CGI-S) [98]. However, nightmares were not the unique target of quetiapine, and the sample included only veteran inpatients with psychosis. In contrast, the 72 VAMC participants were outpatients without significant psychosis who were treated with a broader dosing range (12.5–800.0 mg/day) of quetiapine. Literature supporting the use of quetiapine for PTSD nightmares is limited. Davidson et al. [19] and Aurora et al. [21] both cited an open-label study of quetiapine (25–300 mg) with favorable results for 20 veterans as measured by the CAPS, Positive and Negative Syndrome Scale (PANSS), and HRSD scales [89]. Van Liempt et al. [20] cited an open-label quetiapine (25–300 mg) study that reduced nightmares in 25 combat veterans with PTSD [53]. Results showed limited effectiveness even though the dose range was expanded to 800 mg per day. There were as many trial failures (50%) as partial (41.7%) and fully successful (8.3%) trials combined. The quetiapine dose range in the studies cited by Davidson et al. [19] and van Liempt et al. [20] was 25–300 mg, with encouraging results. VA clinicians raised the upper limit to 800 mg, which resulted in as many failed trials as successful trials.

Of the antipsychotics prescribed for limited participants [3,4,5] in the VAMC study, olanzapine (100%), perphenazine (75%), and ziprasidone (66.7%) had the most favorable outcomes. VAMC clinicians prescribed olanzapine in only five trials using a dose range of 2.5 mg to 10.0 mg. It stopped nightmares in one trial (5 mg) and reduced symptoms in the other four trials (2.5–10 mg). Van Liempt et al. [20] and Aurora et al. [21] cited another study where olanzapine 10–20 mg was reported to be useful in augmenting SSRIs, mood stabilizers, benzodiazepines, and typical antipsychotics in five treated veterans who were resistant to selective serotonin reuptake inhibitors (SSRIs) [52]. However, olanzapine was not utilized as a dedicated agent to treat nightmares [52]. As a dedicated anti-nightmare agent, olanzapine is not widely investigated. Davidson et al. [19] cited one case in which olanzapine augmented an SSRI [91] and double-blind placebo-controlled trial [93]. In the first, 15 mg of olanzapine modifying an SSRI improved sleep and PSQI scores, but there was no change in CGIC scores [91]. Davidson et al. [19] and Aurora et al. [21] rated olanzapine as having insufficient evidence for its use as a dedicated PTSD nightmare medication. Currently, olanzapine is not FDA approved for the treatment of PTSD nightmares [84]. Based on limited data, additional olanzapine trials would help determine olanzapine’s role as a dedicated PTSD nightmare medication.

Some atypical antipsychotics have had limited use in the treatment of PTSD nightmares. Neither Davidson et al. [19] or Van Liempt et al. [20] cited any studies for aripiprazole and ziprasidone. Aurora et al. [21] reported one study in which aripiprazole (15–30 mg) improved nightmares in four out of five participants [24]. In the VAMC study, only four VA veterans received aripiprazole for nightmares. It failed to decrease or extinguish PTSD nightmares in three trials (dose range 5.0–10.0 mg, longest trial 753 days) and prompted a partial response in one trial (dose range 40–80 mg, trial length 1095 days).

In its limited use at the VAMC, aripiprazole had three failures and one partial response within a dosing range of 5.0–10.0 mg. A chart review of 27 treatment resistant veterans with military-related PTSD [99] and a 16-week open-label trial of aripiprazole 9.6 ± 4.3 mg/day with 32 civilians with PTSD recorded reduction of symptoms [100]. The aripiprazole dosing range in the latter study was relatively similar to that utilized by the VAMC clinicians. As with most studies employing antipsychotics for PTSD, the agents were not dedicated to treating nightmares. Other studies for the use of aripiprazole for PTSD nightmares were not found.

Ziprasidone was not reviewed by Davidson et al. [19], Van Liempt et al. [20], or Aurora et al. [21]. In three VA trials, ziprasidone failed in one case (dose 40 mg, trial length 90 days) and achieved a partial response in two trials (40–80 mg, longest trial 426 days). Literature searches did not reveal any other studies for the use of ziprasidone for PTSD nightmares. Both aripiprazole and ziprasidone have had minimal trials with mixed results as a dedicated agent for PTSD nightmares. More trials are required to justify these antipsychotics as specific agents for nightmare suppression.

### 4.3. Typical Antipsychotics

In the past, typical antipsychotics such as thioridazine and perphenazine have demonstrated some effectiveness for PTSD symptoms such as nightmares and flashbacks [54,101]. Thioridazine reduced the severe flashbacks of a 44-year-old Vietnam combat veteran [54]. Thioridazine was noted by Van Liempt et al. [20] to have anecdotal evidence for use in PTSD nightmares. It was not utilized by VA clinicians for nightmares. No English scientific studies of perphenazine as an anti-nightmare were found. Perphenazine was not rated by the three review authors [19,20,21], and it was the only typical antipsychotic chosen for PTSD nightmare suppression at the VAMC. In the four trials reviewed, perphenazine had one full response at 8.0 mg and two partial responses at dose ranges of 4.0 to 8.0 mg. The single failure was at the low dose of 2.0 mg. The paucity of studies and low number of VA subjects treated place thioridazine and perphenazine in the category of needing more evidence to establish their role in PTSD nightmare treatment.

### 4.4. Antidepressants

Although antidepressants have been used sparingly as dedicated agents for nightmare treatment, trazodone has been shown to be effective for PTSD nightmares in several studies. In one OLT study cited by Davidson et al. [19], trazodone at 400 mg reduced CAPS score and improved sleep (CGI) for six participants experiencing PTSD nightmares [102]. Both Van Liempt et al. [20] and Aurora et al. [21] cited the study of Warner et al. [61], in which trazodone (50–200 mg) decreased nightmares (3.3 to 1.3 nights/week, *p* < 0.005) and improved sleep in 72 percent of 60 patients during an eight-week trial. Hoverer, the adverse side effect of priapism (12 percent) occurred more frequently than predicted [61]. In another study of veterans with symptoms of PTSD and sleep disturbance, 42 percent of veterans under 60 and 50 percent of veterans over 60 had positive responses to trazodone. The authors posited that the response was due to deepened non-REM sleep early in the night as well as delayed REM-sleep onset [103]. VA clinicians employed trazodone in 22 trials for nightmare suppression. It was ineffective in two-thirds of the trials with a dose range of 50–200 mg. There were seven partial responses with an expanded dose range of 50–300 mg and one full response using a dose of 200 mg. The overall findings suggest that the effective dosing range for trazodone use as a PTSD nightmare agent is very broad, from 50.0 to 400.0 mg.

Mirtazapine, a selective α2-adrenergic blocker, was prescribed by VA clinicians for PTSD nightmares in 20 trials. Results were mixed as 50 percent of the trials failed and 50 percent achieved partial or full response. The dose range for the 10 failed trials was 7.5–30.0 mg. Nine trials achieved a partial response (dose range 7.5–30.0 mg). One full response was recorded at 15.0 mg; however, the medication treatment was only for five days. Higher dosing at 45.0 mg, as employed by [22], may have improved outcomes. Davidson et al. [19] noted that mirtazapine 45 mg was effective in reducing PTSD symptoms in two studies with a total of 45 subjects. However, it was not prescribed specifically to treat PTSD nightmares [22,104]. Van Liempt et al. [20] cited one 300-participant OLT study in which nightmares and insomnia were improved [105]. Aurora et al. [21] did not cite any mirtazapine studies. Notably, there have been isolated reports of mirtazapine provoking nightmares [106]. Although research regarding the use of mirtazapine for PTSD nightmares is scarce, the VA Office of Research and Development has an ongoing eight-week randomized, double-blind, placebo-controlled treatment trial of mirtazapine for the treatment of PTSD, with dosing ranges from 15 mg to 45 mg/night [107].

Nefazodone was prescribed by VAMC providers twice at doses of 50–200 mg and achieved partial responses. Davidson cited a DPCT study by Davis et al. [108] with 26 nefazodone subjects and 15 controls. The nefazodone treatment group had improved total scores from baseline compared with those in the placebo group (*p* = 0.04 at effect size of 0.6). Hamilton Depression Scale (HAMD) showed significant improvement compared with the placebo (*p* = 0.008). The nefazodone group also reported improvement on the PTSD checklist (*p* = 0.008). Van Liempt et al. [20] and Aurora et al. [21] collectively cited eight OLT studies with a total of 198 participants using nefazodone doses of 386 mg–600 mg (±192 mg) to improve nightmares and insomnia [55,56,57,58,59,60,109,110]. It can be suggested, based on other studies, that higher dosing may have improved results VAMC treatment outcomes [55,57,58]. Studies suggest that nefazodone is an effective medication for PTSD nightmares at doses of 50–600 mg. However, the risk of hepatotoxicity has removed nefazodone from most clinicians’ armory for treating PTSD nightmares.

Several antidepressants were utilized sparingly for PTSD nightmares by the VAMC clinicians. These include paroxetine, citalopram, and sertraline. All three medications were prescribed only once. Paroxetine and citalopram cases both resulted in partial success for the patients, at doses of 60 mg and 40 mg, respectively. The three review authors did not cite any studies employing these drugs as dedicated agents for the treatment of PTSD nightmares [19,20,21].

Studies using sertraline for PTSD nightmares were cited by each of the three review authors [19,20,21]. Davidson et al. [19] noted that sertraline was shown to be effective in preventing the relapse of post-traumatic stress disorder in three large double-blind, placebo-controlled studies with a total of 409 subjects [111]. Van Liempt et al. [20] cited another study by Davidson [19] (*n* = 208), and Aurora et al. [21] reported a small study by Lambert [24] with five subjects. Sertraline has been shown to be effective in preventing the relapse of posttraumatic stress disorder in three large double-blind, placebo-controlled studies with a total of 409 subjects [111,112,113]; however, PTSD nightmares were not specifically targeted. VA treatment teams only used sertraline in one unsuccessful trial with a maximum dose of 200 mg. At this time, studies do not support sertraline as an effective dedicated treatment for PTSD nightmares.

### 4.5. Tricyclic Antidepressants

Tricyclic antidepressants (TCAs) are a class of antidepressants that inhibit the reuptake of norepinephrine or serotonin at presynaptic neurons. They have been prescribed for PTSD nightmares, but with limited success [19]. Davidson et al. [19] reported two early TCA studies with veterans having PTSD [70,114]. In one, the 46 veterans did not have a positive response to amitriptyline, and PTSD nightmares were not targeted as a unique outcome measure [70]. The second study involved an imipramine–placebo-controlled study of 23 veterans with PTSD. Kinzie and Leung [82] reported mixed results in an OLT study utilizing a combination of clonidine and imipramine in 68 severely traumatized Cambodian refugees with PTSD and major depression. In this study, PTSD nightmare frequency decreased in seven patients.

Aurora et al. [21] classified amitriptyline, clomipramine, and nortriptyline as marginally effective for PTSD nightmares. They cited a small open-label study with 12 subjects [70] in which nightmares improved in 10 subjects while utilizing a variety of TCAs as adjunctive and monotherapy: (1) monotherapy: imipramine, doxepin, amitriptyline; (2) adjunctive TX: imipramine + phenelzine; doxepin + phenelzine, doxepin + amitriptyline). PTSD symptoms improved but nightmares were not identified as a unique treatment outcome. VA providers utilized a TCA in only one trial. Amitriptyline 50 mg achieved a partial PTSD nightmare response. There were limited encouraging studies cited in the three reviews utilizing tricyclics [25,69,70,82,114], but these and other TCA studies are limited, old, and inconclusive regarding the appropriate use for PTSD nightmare treatment.

### 4.6. Benzodiazepines

Benzodiazepines were noted early in the treatment of PTSD to have the disadvantages of tolerance and addiction in PTSD veterans [115]. However, evidence for the use of benzodiazepines in veterans with PTSD has been controversial [116]. Reduced GABA levels have been associated with PTSD [117]. They may also play a role in the symptomatic relief of irritability, sleep disturbances, and other hyperarousal symptoms. GABA-A transmission increases presynaptic inhibition in the limbic system, thalamus, and hypothalamus, thus promoting short-term relief of anxiety symptoms [84].

A 40-participant crossover study with triazolam for 40 participants reduced unpleasant dreams [74]. Davidson et al. [19] reviewed studies of alprazolam [73], clonazepam plus alprazolam (Gelpin 1996) [118], and temazepam [119], all of which did not improve PTSD nightmares. Van Liempt et al. [20] cited studies of temazepam [72] and clonazepam [71], which also did not improve PTSD nightmares or insomnia. There were only three trials of benzodiazepines for PTSD nightmares in the VAMC experience. Partial responses were recorded for clonazepam (1.0 mg) and temazepam (15.0 mg). The single benzodiazepine trial that had a full response in muting PTSD nightmares employed lorazepam 1.0 mg.

It has been reported that veterans with PTSD are frequently prescribed benzodiazepines that are not supported by existing evidence and current guidelines [120]. The use of benzodiazepines has not been demonstrated to improve PTSD symptoms [11,73,121]. Significantly, the VA and Department of Defense clinical guidelines specifically do not recommend benzodiazepine use in the treatment of PTSD [122].

### 4.7. Sulfamate-Substituted Monosaccharaide Antiepileptics

Topiramate was the only sulfamate-substituted monosaccharaide antiepileptic prescribed by the VAMC clinicians for PTSD nightmares. It has been assessed as having limited success in the treatment of PTSD nightmares [19,20,21]. VA providers prescribed topiramate only twice with mixed success utilizing doses within the dosing range of Berlant 2002 (12.5–500.0 mg per day) [31]. A VAMC 30-day trial using 100 mg was a failure, while a dose of 200 mg/day improved another veteran’s PTSD nightmares. Berlant published three studies of topiramate for nightmares [31,32]. In the first study, topiramate doses from 12.5 to 500 mg/day were employed both as add-ons (*n* = 28) and as monotherapy (*n* = 7). Nightmares decreased in 79 percent (19/24) of the civilian subjects with PTSD; however, there was an unexpectedly high dropout rate [31]. In a second study, topiramate (five monotherapy, 28 augmentation) reduced PTSD symptoms from baseline to four weeks in civilians, as measured by the PTSD Checklist-Civilian Version PTSD [32]. In another Berlant report [32], topiramate decreased nightmares in five monotherapy and 28 augmentation trials. Based on PTSD Checklist-Civilian Version (PCL-C) scores, total symptoms declined by 49 percent at week 4 [32]. Berlant cautioned that analysis of three small, double-blind, placebo-controlled clinical trials were not problem-free, including the unexpectedly high dropout rates in one study. Thus generalization from these initial studies requires future adequately powered clinical trials. Most of the research of topiramate for PTSD was with civilians without a focus on nightmares. There is initial evidence to support the further study of topiramate for treating PTSD nightmares in veterans.

### 4.8. Medication Combinations for PTSD Nightmare Treatment

Partial response usually requires increasing monotherapy to a higher or maximal dosage. If the medication is at the highest recommended dose, augmentation may be employed. Augmentation may boost the effectiveness of the single agent to an optimal clinical response. However, the disadvantages of augmentation include increased risk of adverse side effects and drug interactions [123]. VAMC clinicians’ efforts to utilize medication combinations dedicated specifically for PTSD nightmare reduction were relatively infrequent and marginally successful. Of the 22 trials recorded, 17 (77.3%) were unsuccessful, three (13.6%) achieved partial responses, and one (0.5%) had a full response. Prazosin (2.0–4.0 mg) plus trazodone (50–200 mg) achieved partial responses (0.5%) with the longest trial, lasting 460 days. Two combinations of medications resulted in full suppression of nightmare recall in two trials: prazosin (1.0 mg) plus risperidone (2.0 mg) were successful (30 day trial); and mirtazapine (7.4 mg) plus risperidone (1.5 mg) extinguished nightmare recall for 1346 days in the second case.

Only three of 22 medication combination trials achieved a partial or full response in treating PTSD nightmares. Medication combinations are infrequently reported for improving PTSD nightmare symptoms. There is presently little evidence to support the effectiveness of medication combination treatment.

### 4.9. Medications Not Used in the VAMC Study

There were several drugs reported in other studies that were not prescribed at the VAMC or the WTC, including the following: the non-benzodiazepine imidazopyridine hypnotics of zolpidem, eszopiclone, and zaleplon; the nipecotic acid derivative tiagabine; the anxiolytic buspirone; the anticonvulsant gabapentin; the monoamine oxidase inhibitor phenelzine; and the glucocorticoid cortisol. In addition, the antidepressants venlafaxine, fluvoxamine, fluoxetine, and nefazodone were not prescribed by the VAMC providers. These appeared to be somewhat less frequently successful than observed with the VAMC-prescribed medication, and some may also entail undesired medical risks.

In general, benzodiazepine-like sedative-hypnotics may promote dissociative episodes and worsen PTSD nightmares [124]. Zolpidem has the advantage of rapid induction of sleep, short duration, and low tolerance and addiction risk. In addition to having few adverse medication interactions, Zolpidem was the only non-benzodiazepine imidazopyridine prescribed for PTSD nightmares that was cited by both Davidson et al. [19] and van Liempt et al. [20]. Dieperink and Drogenmuller [125] reported a reduction of PTSD nightmares in their small trial with zolpidem 10 mg. Davidson et al., Van Liempt et al., and Aurora et al. [19,20,21] did not review eszopiclone or Zaleplon. A more recent study by Pollack et al. suggests that 3 mg of eszopiclone can lessen the severity of PTSD nightmares in the short term, as well as improve sleep when compared with the placebo [29]. Presently, there is scarce evidence to support using non-benzodiazepine imidazopyridine hypnotics such as zolpidem, eszopiclone, and zaleplon for PTSD nightmares.

Tiagabine, a nipecotic acid derivative, improves sleep by decreasing the reuptake of GABA [126,127]. It enhances slow wave sleep and sleep maintenance in primary insomnias [128]. Two tiagabine studies were cited by Davidson et al. and Van Liempt et al. [19,20]. Tiagabine doses from 4–12 mg per day [34] improved PCL-C scores within two weeks for six of the seven female subjects. Nightmares were not specifically targeted. In a multicenter study of tiagabine, 232 patients were randomized (tiagabine 116; placebo 116) [35]. Dosing was titrated from 4 mg per day weekly to a maximum dose of 16 mg per day. The Clinician-Administered PTSD Scale total score revealed no significant differences (*p* = 0.85) from baseline utilizing tiagabine at the final visit when compared with the placebo. The authors concluded that tiagabine was not significantly different from the placebo in the treatment of symptoms of PTSD.

Van Liempt et al. [20] cited one small study of the anxiolytic buspirone (*n* = 3) by Wells [36] that demonstrated an improvement in insomnia with doses from 35 to 60 mg per day; PTSD nightmares were not targeted. Limited evidence does not support the use of buspirone for treating PTSD nightmares.

A study by Hamner et al. [37] of the anticonvulsant, gabapentin, for the treatment of PTSD symptoms was cited by both Van Liempt et al. and Aurora et al. [20,21] as an alternative PTSD nightmare medication. In an OLT study of 30 consecutive PTSD patients, 77 percent had a decrease in the nightmare frequency within a wide dose range of 300 to 3600 mg per day. The authors noted that the use of anticonvulsants in PTSD may be problematic as the cytochrome P450 pathway may displace other protein-bound medications [37]. VA clinicians did not prescribe gabapentin for PTSD nightmare suppression. Over a wide dosing range, gabapentin demonstrated some initial effectiveness for treating PTSD nightmares.

The monoamine oxidase inhibitor phenelzine is an antidepressant that began to be utilized for the treatment of PTSD symptoms including PTSD nightmares in 1982. Davidson et al. [19] cited two RPCT studies by Kosten et al. [69]. Of the 19 participants prescribed phenelzine, there was a 44 percent reduction of PTSD symptoms on the IES Scale [69]. Van Liempt et al. [20] noted three studies in their nightmare medication review. Hogben et al. [38] evaluated five subjects with PTSD who had failed multiple previous therapeutic trials with antipsychotics, tricyclic antidepressants, and psychotherapy with or without medication. Disturbing episodes of nightmares and flashbacks improved with phenelzine. Lerer et al. conducted an open-label trial of phenelzine (30–90 mg) with 25 Israeli combat veterans [39]. Although seven of the 12 items on the PTSD scale improved, only the sleep index showed a significant improvement. Phenelzine was not cited by Aurora et al. [21], nor was it used by VA practitioners for PTSD nightmare treatment. Phenelzine has had limited clinical trials with mixed results in treating PTSD nightmares.

The administration of glucocorticoids may reduce the retrieval and vividness of the traumatic PTSD memories [40]. Cortisol was not included in the Davidson et al. or Van Liempt et al. [19,20] reviews. Aurora et al. [21] cited one small study [129] in which three combat veterans experienced a 38 percent reduction in the daily symptoms of traumatic memories after receiving low-dose cortisol (10 mg/day) orally for one month. Presently, there is little evidence to support cortisol as a specific agent for improving PTSD nightmare symptoms.

The several studies of the antidepressant fluvoxamine for PTSD nightmare treatment have had mixed results [62,63,64]. Davidson et al. [19] cited a small study of combat veterans that did not target nightmares, but reduced PTSD symptoms except for depression (CAPS, HAMD) [62]. Van Liempt et al. [20] cited a study by Neylan of 21 Vietnam veterans whose PTSD nightmares improved on a modal dose of 150 mg (range 100–250 mg) during this 10-week open-label study [63]. Aurora et al. [21] cited a 12-week study of 24 Dutch World War II resistance veterans with chronic PTSD who had their combat nightmares, chronic insomnia, anxiety, intrusive recollections, feelings of guilt, and tiredness improved with fluvoxamine doses [64]. Although fluvoxamine was not used by the VA clinicians for PTSD nightmares, some preliminary evidence supports its use in treating PTSD nightmares by employing doses from 100 to 250 mg.

In the revised VA guidelines, the antidepressant venlafaxine has become a first-line PTSD agent [130]; however, it has not been recommended specifically as a PTSD nightmare treatment. Large DPCT venlafaxine studies were reviewed by Davidson et al. [19]. CAPS assessments showed improvement in PTSD symptoms; however, venlafaxine was not specified as a monotherapeutic agent for PTSD nightmares. Van Liempt et al. [20] did not cite venlafaxine. It was not recommended for PTSD nightmare treatment by Aurora et al. [21]. In a more recent large study with 340 subjects by Stein et al. [65], there was no improvement of nightmares using venlafaxine. Venlafaxine was not used by the VAMC physicians for nightmare reduction, and it does not appear to be an effective treatment option for PTSD nightmare treatment.

The antidepressant fluoxetine was not used by VA clinicians for PTSD nightmares; however, it has been found to be partially effective in some cases. Davidson et al. [19] cited a controlled study [68], a double-blind, randomized study [22], and a double-blind, randomized, placebo-controlled study (Martenyi 2002 [67] with a total of 186 participants. While PTSD symptoms were reduced, PTSD nightmares were not the primary treatment objective. Van Liempt et al. [20] cited a study with 53 participants in which there was no change in the nightmare symptoms [66]. A more recent study has shown fluoxetine to be effective for PTSD symptoms [84]. However, there do not appear to be adequate studies with fluoxetine to support its use as a dedicated medication for PTSD nightmares.

## 5. Limitations

As a retrospective study without controls, this study can only assess the effectiveness of medications prescribed for PTSD nightmares in a VAMC on a non-experimental basis. The intention was to gauge the VA clinicians’ use of medications compared to clinical trials reviewed by Davidson et al., Van Liempt et al., and Aurora et al. [19,20,21]. The study was not able to include many of the results of VAMC’s pharmacologic interventions because of insufficient data pertaining to treatment outcome. Clinicians also often failed to indicate in the chart whether the medications prescribed for PTSD symptoms were specifically for nightmare treatment, even in cases where a change in nightmare status was recorded. Also, the treatment response was graded at three levels (no response, partial response, or full response) rather than citing results based on assessment scales such as the Clinician-Administered PTSD Scale (CAPS), total CAPS score, Clinical Global Impressions Severity Scale (CGI-S), Clinical Global Impressions Change Scale (CGI-C), the Mississippi Scale for Combat-Related PTSD, and the Combat Exposure Scale (CES). Despite these limitations, the results in this survey on actual prescribing patterns contain useful information about the effectiveness of previously tested medications for PTSD combat nightmares and suggest avenues for further study in clinically controlled trials of several medications.

## 6. Conclusions

A limited number of medications have been employed as dedicated agents for PTSD nightmares in active duty soldiers and veterans. This is the first study of open-label use of mediations dedicated to the treatment of PTSD nightmares at a VAMC. Studies exploring the use of monotherapy specifically for PTSD nightmares are limited. Most studies discuss the use of medications for the spectrum of PTSD symptoms, rather than for the individual symptoms that are most disturbing to the patient. Multiple studies have involved many of the medications utilized by the VAMC for PTSD nightmare treatment. The majority of medications for PTSD have focused on treatment of the full or partial spectrum of PTSD symptoms. For the most part, the dosing ranges chosen by the VA clinicians represented the reported dosing ranges of the studies cited in three prior reviews of PTSD nightmare medications prescribed specifically for nightmare resolution [19,20,21]. Furthermore, except where noted, the success rates were similar to the trials in those studies.

Regarding several medications, some differences should be mentioned. For the medications prescribed for more than 50 veterans, prazosin failed slightly more than it was successful at the same dosing range used in the successful studies of the three reviewers. Risperidone was the most successful VAMC agent in terms of number of prescriptions and success, but with a higher upper dose limit than in other cited studies. With an upper limit twice that cited in other studies, quetiapine had a 50 percent success rate. Also, of medications utilized for between six and 50 veterans, VAMC clinicians successfully prescribed clonidine at a broader dosing range than cited by the three reviewers. Terazosin, which has not been investigated extensively or cited in the reviews, proved effective in more than 50 percent of the cases. Poor results were recorded for trazodone using a dosing similar to other studies. Of those medications prescribed five times or fewer and not represented in other studies, olanzapine was the most notable, with 100 percent effectiveness using a modest dosing range.

Overall, for those medications with approximately 50 percent success or better for VAMC clinicians, three had a higher dosing limit and three had similar dosing limits for treating PTSD nightmares compared to the successful studies cited by the three reviewers. In general, it appears that the upper dosing limits of many agents have not been investigated, as with prazosin, and in further treatment these doses should be modified accordingly. The findings of this comparative study strongly suggest the need for additional investigation of multiple medications. In addition, the positive results for risperidone at this VA could suggest its use as the first treatment choice for PTSD nightmares, since positive results occur rapidly at a limited dose range. However, this choice would need to be weighed against the only moderately less successful clonidine and terazosin and their lower risk of adverse effects. Further study of this option is warranted.

## Figures and Tables

**Table 1:** Single and combinations of medications utilized to treat combat nightmares.

**Medications**	**Number of Trials**	**No Responses**	**Partial Response**	**Full Response**	**Total Success**
**Alpha 2 Adrenergic Receptor Agonists**					
Prazosin (Aurora 2010 ber/percent) Dose range (mg) Maximum Treatment range (days)(avg)	106	54 (51%) −20.0 (1–2005)	46 (43%) −10.00 (7–2222)	6 (6%) −10.0 (12–2003)	49%
Clonidine (number/percent) Dose range (mg) Maximum Treatment range (days)(avg)	27	10 (37%) −4.0 (2–1087)	17 (63%) 0.1–2.0 (21–1216)	0	63%
Terazosin (number/percent) Dose range (mg) Maximum Treatment range (days)(avg)	26	10 (39%) 1.0–20.0 (8–1995) 551	16 (61%) −50.0 (21–2190)	0	61%
**Atypical Antipsychotics/Benzisoxazole derivatives**					
Risperidone (number/percent) Dose range (mg) Maximum Treatment range (days)(avg)	81	19 (23%) 0.25–5.0 (3–1580)	41 (51%) 0.5–6.0 (6–2175)	21 (26%) 0.5–3.0 (5–2615)	77%
Quetiapine (number/percent) Dose range (mg) Maximum Treatment range (days)(avg)	72	36 (50%) 12.5–500.0 (1–2527)	30 (42%) 25.0–800.0 (2–2520)	6 (8%) 12.5–50.0 (6–287)	50%
Olanzapine (number/percent) Dose range (mg) Maximum Treatment range (days)(avg)	5	0	4 (80%) 2.5–10.0 (78–1170) 410	(20%) 5.0 105	100%
Aripiprazole (number/percent) Dose range (mg) Maximum Treatment range (days)(avg)	4	(75%) 5.0–10.0 (7–2100)	1 (25%) 10.0 1095	0	25%
Ziprasidone (number/percent) Dose range (mg) Maximum Treatment range (days)(avg)	3	1 (33.3%) 40 90	2 (67%) 40.0–80.0 (365–486)	0	67%
**Typical Antipsychotics**					
Perphenazine (number/percent) Dose range (mg) Maximum Treatment range (days)(avg)	4	1 (25%) 2.0 120	2 (50%) −8.0 (325–485)	1 (25%) 8.0 257	75%
**Antidepressants**					
Trazodone (number/percent) Dose range (mg) Maximum Treatment range (days)(avg)	22	14 (64%) 50.0–200.0 (2–4375)	7 (32%) 50.0–300.0 (231–1395)	1 (4%) 200.0 295	36%
Mirtazapine (number/percent) Dose range (mg) Maximum Treatment range (days)(avg)	20	10 (50%) 7.7–30.0 (10–1326)	9 (45%) 7.5–30.0 (48–5630)	1 (5%) 15.0 5	50%
Medication Combinations Utilized to Treat Combat Nightmares					
**Medications**	**Number**	**No Responses Number of Responses, Dose Range, Length of Treatment (Days)**	**Partial Response Number of Responses, Dose Range, Length of Treatment (Days)**	**Full Response Number of Responses, Dose Range, Length of Treatment (Days)**	**Total Success**
prazosin + trazodone (number/percent) Dose range (mg) Maximum Treatment range (days)	5	3 (60%) 2.0–10.0 + 50–100 (2–1152)	2 (40%) 2.0–4.0 + 50–200 (169–750)	0	40%
prazosin + quetiapine (number/percent) Dose range (mg) Maximum Treatment range (days)	5	4 (80%) 1.0–5.0 + 25–450 (13–364)	1 (20%) 2.0–25 187	0	20%

**Table 2:** Veterans Affairs Medical Center dose range and dose mode response for the seven most prescribed medications for combat nightmares.

Medication	Number No Responses	Dose Range (mg) No Responses	Dose Mode (mg) No Responses	Number Partial + Full Responses	Dose Range (mg) Partial + Full Responses	Dose Mode (mg) Partial + Full Responses
**Atypical Antipsychotics/Benzisoxazole Derivatives**						
Prazosin	54	1.0–20.0	2.0	51	1.0–10.0	2.0
Clonidine	10	0.1–4.0	0.1	17	0.1–2.0	0.1
Terazosin	9	1.0–20.0	1.0	16	2.0–50.0	2.0
**Atypical Antipsychotics/Benzisoxazole Derivatives**						
Risperidone	19	0.25–5.0	2.0	72	0.25–6.0	2.0
Quetiapine	36	12.5–500.0	25.0	36	12.5–800.0	50.0
**Antidepressants**						
Trazodone	8	50.0–200.0	100.0	14	50.0–300.0	100.0
Mirtazapine	10	7.5–30.0	30.0	10	7.5–30.0	15.0

**Table 3:** Combat nightmares: successful medication dose ranges in three literature reviews.

Medications	Davidson et al. 2005 [19]	Van Liempt et al. 2006 [20]	Aurora et al. 2010 [21]	Veterans Affairs Medical Center Full+ Partial
**Alpha 2 Adrenergic Receptor Agonists**				
Prazosin Number Dose range (mg)	Raskind 2002 [44] 59 5.5–10.5	Raskind 2000 [45] 4 2–5	Taylor 2002 [34] 5 1–4	52 1.0–10.0
		Raskind 2002 [44] 59 8.7–10.5	Peskind 2003 [46] 9 2–4	
	Raskind 2003 [46] 10 9.5			
		Taylor 2002 [34] 5 1–4	Raskind 2003 [46] 10 9.5	

		Raskind 2003 [46] 10 9.5	Raskind 2007 [26] 40 10.3–16.3	
		Peskind 2003 [46] 9 2–4	Taylor 2008 [47] 13 2–6	
Clonidine Number Dose range (mg)		Kinzie 1994 [48] 4 0.3		17 0.1–2.0
Terazosin				16 2.0–50.0
Cyproheptadine Number Dose range (mg)		Brophy 1991 [49] 4 16–24	Brophy 1991 [49] 4 16–24	Not prescribed
		Gupta 1998 [50] 9 4–12		
**Atypical Antipsychotics/Benzisoxazole derivatives**				
Olanzapine Number Dose range (mg)		Stein 2002 [51] 19 10–15		5 2.5–10.0
Risperidone Number Dose range (mg)		Leyba 1998 [52] 4 1–3		62 0.25–6.0
Quetiapine Number Dose range (mg)		Robert 2005 [53] 20 25–300		36 12.5–800
Aripiprazole Number Dose range (mg)			Lambert 2006 [24] 5 15–30	1 10.0
Ziprasidone Number Dose range (mg)				2 40–80
**Typical Antipsychotics**				
Perphenazine Number Dose range (mg)				34.0–8.0
Thioridazine Number Dose range (mg)	None	Dillard 1993 [54] 1	None	None
**Antidepressants**				
Paroxetine				1 60
Citalopram				1 40
Sertraline				1 0
Mirtazapine				10 7.5–30.0
Nefazodone Number Dose range (mg)		Davidson 1998 [55] 17 200–600	Davidson 1998 [55] 17 200–600	2 30–200
		Hidalgo 1999 [56] 105 400–600	Gillin 2001 [57] 12 441	
			Neylan 2003 [58] 10 400–600	
		Zisook 2000 [59] 19 100–600		
		Gillin 2001 [57] 12 441		
		Neylan 2003 [58] 10 400–600		
Trazodone Number Dose range (mg)		Hertzberg 1998 [60] 6 400	Warner 2001 [61] 74 25–200	8 50–300
Fluvoxamine Number Dose range (mg)	Escalona 2002 [62] 14	Neylan 2001 [63] 21	Neylan 2001 [63] 21	None
		De Boer 1992 [64] 24	De Boer 1992 [64] 24	
Venlafaxine Number Dose range (mg)	Davidson 2005 [19] 329	None	Stein 2013 [65] 339	None
	Davidson 2005 [19] 538			
Fluoxetine Number Dose range (mg)	Connor 1999 [22] 53	Meltzer-Brody 2000 [66] 52	None	None
	Martenyi 2002 [67] 69			
	van der Kolk 1994 [68] 33			
		Tricyclic Antidepressants		
Amitriptyline	None	None	None	1 50
Imipramine Number Dose range (mg)	Kosten 1991 [69] 23		Boehnlein 1985 [70] 12	None
Doxepin Number Dose range (mg)	None	None	Boehnlein 1985 [70] 12	None
		Benzodiazepines		
Clonazepam Number Dose range (mg)		Cates 2004 [71] 6 1–2	Cates 2004 [71] 6 1–2	1 1.0
Temazepam Number Dose range (mg)		Mellman 2002 [72] 22 0 mg × 5 days 15 mg × 2 days stop		1 15
Lorazepam Number Dose range (mg)	Connor 1999 [22] 53			1 1.5
	Martenyi 2002 [67] 69			
	van der Kolk 1994 [68] 33			
Alprazolam Number Dose range (mg)	Braun 1990 [73] 10	None	None	None
Triazolam Number Dose range (mg)	None	None	Ellingsen 1983 [74] 40	None
Nitrazepam Number Dose range (mg)	None	None	Ellingsen 1983 [74] 40	None
**Sulfamate-Substituted Monosaccharide Antiepileptics**				
Topiramate Number Dose range (mg)	Berlant 2002 [31] 35 12.5–500	Berlant 2002 [31] 35 12.5–500	Berlant 2002 [31] 35 12.5–500	1 200
Monoamine Oxidase Inhibitor (MAOI)				
Phenelzine	None	Hogben 1981 [38] 5	None	None
		Lerer 1987 [39] 25		

## Abbreviations

The following abbreviations are used in this manuscript:

PCT	Placebo-controlled trial
RPCT	Randomized placebo-controlled trial
DRPCT	Double-blind placebo-controlled trial
OLT	Open-label trial
DPCT	Double-blind placebo-controlled trial
HAMD	Hamilton Depression Scale
CAPS	Clinician-Administered PTSD Scale
PANSS	Positive and Negative Syndrome Scale
HDRS	Hamilton Depression Rating Scale (HDRS), abbreviated HAM-D
CGI-S	Clinical Global Impressions Severity Scale
CGI-C	Clinical Global Impressions Change Scale
CES	Combat Exposure Scale
